# The Programme of the Hospital Saturday Fund

**Published:** 1891-07-04

**Authors:** Reginald B. D. Acland

**Affiliations:** Chairman of the Fund


### The Programme of the Hospital Saturday Fund.

By Mr. Reginald B. D. Acland, Chairman of the Fund.

My attention has been called to a criticism which appeared under the above heading in your issue of June 27th, signed "Agricola," and though I cannot, as I certainly have no desire to, object to any criticism of the work of that Fund which is founded on facts, I could have wished that the tone of the criticism had been a little less bitter and a little more courteous, the more so as while your contributor professes to be dealing with the evidence given by me before the Committee of the House of Lords, no opportunity is afforded to your readers of forming any judgment upon my evidence, my name not even appearing among those of the witnesses called; and the most charitable view I can take of the article in question is that even "Agricola" himself has not read the evidence with attention.

With your permission I desire to call attention to one or two statements of fact. "Agricola" suggests that I gave some evidence to the effect "that the authorities of the Fund are bent on exacting a *quid pro quo* in return for any assistance they render." It is, perhaps, sufficient for me to say that I never said or suggested anything of the sort.

What I did say is, "We receive letters of recommendation in proportion to our grants sometimes according to the same scale as that on which they give them to ordinary subscribers, and sometimes according to a special scale." Further, in answer to a question of the Chairman, "What is the definite aim of your Fund?" the receipt of letters of recommendation was not even referred to.

Again, "Agricola" writes: "It (the Hospital Saturday Fund) desires to pose as the censor of the institutions to sap their independence and even to alter their character." That again is untrue and entirely without any foundation as far as my evidence is concerned, and is even opposed to what I did say. What I did say in answer to the question above-mentioned (after referring to the knowledge of the Committee, that in a great number of hospitals some beneficial influence was required) was "It is hoped that by degrees we may *assist* in creating a standard of efficiency and economy which it is impossible for an individual to create, and which possibly a central body of good standing and good position may create," and further than this I most explicitly stated in the very next sentence "The Hospital Saturday Fund are most anxious never to meddle with the internal management of the hospitals further than to decide, of course, whether it will make them a grant," and yet in the face of this distinct statement, "Agricola" enquires "what reason there is in the claim of working class representatives to meddle with the management of the" hospitals?

It is true that, while expressing my own view against the proposal, I mentioned evening attendance of patients as one of the aims of the Fund. The last paragraph of the article I confess I am quite unable to understand. "The preposterous self-sufficiency of the Saturday Fund authorities" exists solely in the imagination of "Agricola," who, to my sincere regret, has attacked a body of men of whose aims, motives, and work he is obviously ignorant. He is, of course, entitled to hold any opinion he pleases as to the desirability of working-class representatives being placed on the Boards of Management of hospitals, but so also may the "authorities of the Saturday Fund" and the hospitals themselves. Many of the latter actually encourage the creation of such a class of life and annual governors—notably the Victoria Park Hospital and others in the East-end of London. But before "Agricola" criticises the scheme of the Hospital Saturday Fund, he should, I think, make himself familiar with it.

As I said in my evidence before the Committee, the Saturday Fund — except by distributing the money it contributes to the medical charities in such a way that those institutions where the largest amount of work is the most economically and efficiently performed receive the largest grants—do not attempt in any way to interfere with the management of any hospital, but they are of opinion (and apparently no small number of the hospitals agree with them) that if one or more working-class representatives were members of the Boards of Management of the different hospitals, they would, by their knowledge of the class for whose benefit those institutions exist, be of considerable assistance in solving some of the questions arising for decision.

